# Upregulation of CD22 by Chidamide promotes CAR T cells functionality

**DOI:** 10.1038/s41598-021-00227-4

**Published:** 2021-10-19

**Authors:** Xin Yang, Qiuxia Yu, Hao Xu, Jianfeng Zhou

**Affiliations:** Department of Hematology, Tongji Hospital, Tongji Medical College, Huazhong University of Science and Technology, NO.1095 Jie Fang Avenue, Wuhan, 430030 Hubei China

**Keywords:** Cancer, Immunology

## Abstract

Treatment failure or relapse due to tumor escape caused by reduction in target antigen expression has become a challenge in the field of CART therapy. Target antigen density is closely related to the effectiveness of CART therapy, and reduced or lost target antigen expression limits the efficacy of CART therapy and hinders the durability of CAR T cells. Epigenetic drugs can regulate histones for molecular modifications to regulate the transcriptional, translational and post-translational modification processes of target agents, and we demonstrated for the first time the role in regulating CD22 expression and its effect on the efficacy of CD22 CART. In this paper, we found that Chidamide promoted the expression of CD22 on the surface of B-cell tumor cells in vitro and in vivo, and enhanced the function of CD22 CART. As for mechanisms, we demonstrated that Chidamide did not affect CD22 mRNA transcription, but significantly increased the expression of total CD22 protein, indicating that Chidamide may upregulate cell surface CD22 expression by affecting the distribution of CD22 protein. In summary, our results suggest that Chidamide may enhance the efficacy of CD22 CART by inhibiting histone deacetylases to regulate post-transcriptional modifications that affect protein distribution to increase the expression of CD22 on the cell surface.

## Introduction

Acute B-lymphocytic leukemia (B-ALL) and B-cell non-Hodgkin's lymphoma (B-NHL) are common hematologic malignancies, accounting for approximately 85% of all hematologic malignancies. With the improvement of diagnosis and treatment strategies, the 5-year survival rate of ALL in childhood has increased to more than 80%, but the long-term survival rate of ALL in adult is only about 40%^1,2^. The prognosis of relapsed or refractory patients is still poor, with long-term survival rates of only 20–40%, and many high-risk cases and special subtypes still lack effective therapeutic options^3,4^. For these patients, chimeric antigen receptor T cell (CART) immunotherapy, which has emerged as a promising therapeutic strategy for hematologic malignancies in recent years, has shown unexpected efficacy, and CART targeting CD19 can induce remission or even cure for a high percent of patients with relapsed or refractory B-ALL or B-NHL^5–7^. However, long-term follow-up data suggest that a proportion of patients relapse after receiving CD19 CART therapy, with targeted antigen loss being a major cause^8,9^. To overcome this challenge, CART targeting CD22 has been developed. CD22 CART can re-induce remission in 50–80% patients who relapse due to loss of target antigen after CD19 CART therapy^10^, but similar to CD19 CART, relapse also occurs after CD22 CART therapy, in which the problem of target antigen loss is again highlighted^11^.

With the advancement of CART therapy, a certain threshold of target antigen density is required to activate CAR has been confirmed in preclinical models^12,13^. In addition, target antigen density is closely related to the efficacy of CART therapy. The lower target antigen density is one of the limitation for CART therapy, and diminished or lost expression of target antigen is adverse for the persistence and efficacy of CART^14,15^. Studies have shown that patients with lower CD22 expression were only able to maintain stable disease (SD) after CD22 CART therapy, whereas patients with relatively higher CD22 expression could achieve complete remission (CR). Moreover, in patients with lower CD22 expression, even increasing the dose of infused CAR T cells and improving the affinity of CAR binding to CD22 targets did not enhance the CART functionality^16^. Therefore, tumor escape due to diminished or loss of target after CART therapy has become an insurmountable obstacle for immunotherapy and a frontier topic for research at this stage.

In recent years, it has been found that epigenetic drugs can regulate the expression of lymphoma cell-associated antigens and have an impact on the efficacy of targeted therapy^17,18^. Chidamide, a domestic histone deacetylase inhibitor (HDACi), has been shown to have the ability to significantly alter the expression of genes in the cell membrane and pericellular environment-related pathways in B-cell lymphoma cell lines^19^. Therefore, we hypothesized that Chidamide could enhance the functionality of CART by modulating the expression of target antigens or rescue disease relapse due to loss of target expression after CART therapy.

## Results

### Chidamide upregulates CD22 expression on the surface of B-cell tumor cell lines and primary cells in vitro

**Figure 1:**
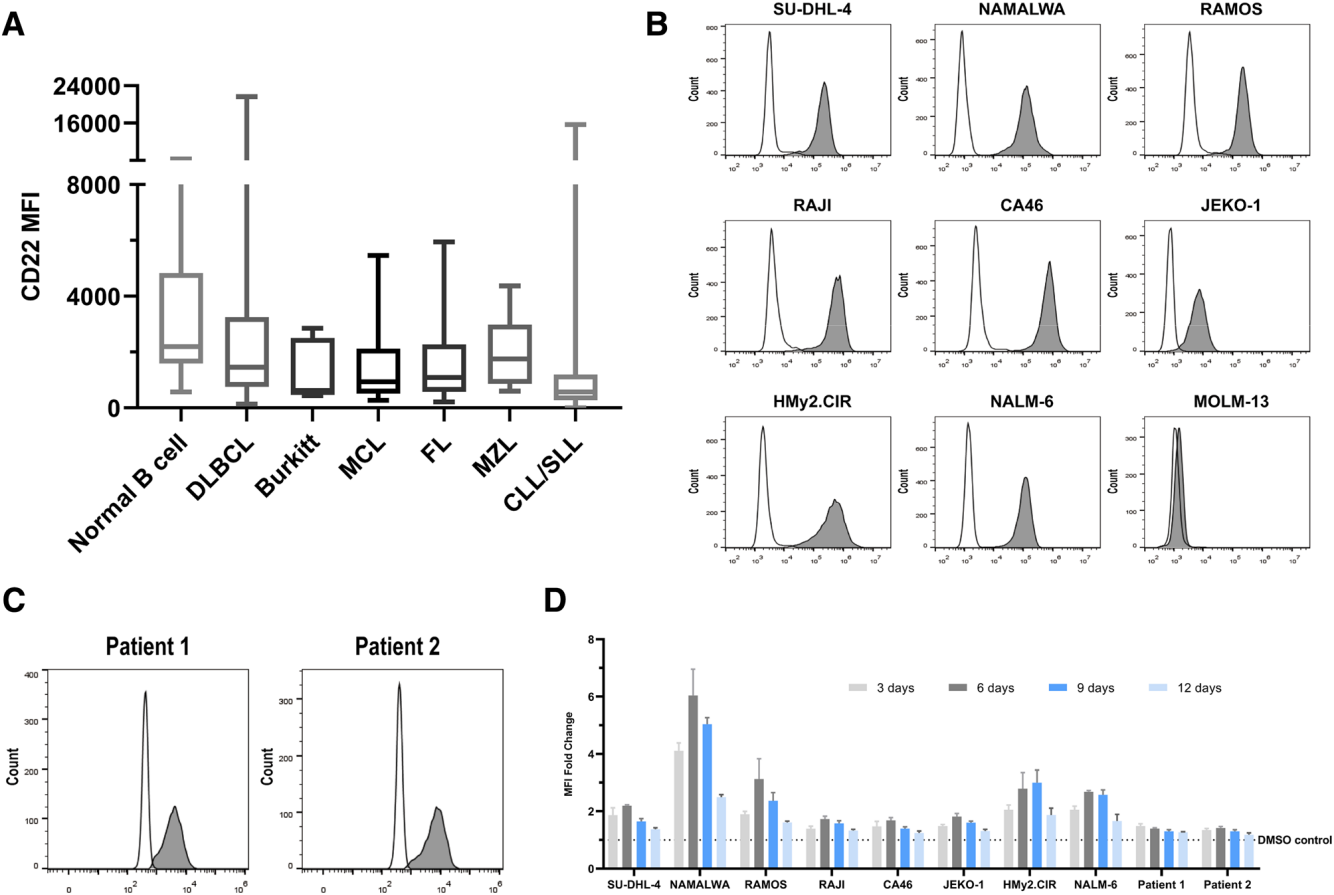
Chidamide upregulates CD22 expression on the surface of B-cell tumor cell lines and primary cells in vitro. (**A**) CD22 expression intensities in diferent B-cell lymphomas. (**B**, **C**) Cell surface CD22 expression in different B-cell tumor lines and the AML cell line MOLM13 as well as 2 cases of patient samples. For all plots, the black line represents staining with the isotype control, and the gray-filled line represents staining with anti-CD22 antibodies. (**D**) Eight cell lines and two primary tumor cells were co-incubated with 0.5 μM Chidamide and analyzed using flow cytometry at different days after Chidamide exposure. MFI fold change = CD22 MFI^Chidamide^/CD22 MFI^DMSO^.

CD22, B-cell differentiation antigen of great significance, is expressed in most B-lineage lymphomas as well as leukemia cells^20,21^. In 227 patients of B-lineage lymphoma hospitalized in the Department of Hematology, Tongji Hospital, Tongji Medical College, Huazhong University of Science and Technology, the expression of CD22 was positive in clinical tests for all specimens (Fig. 1A). In addition, the B-cell tumor cell lines SU-DHL-4, NAMALWA, RAMOS, RAJI, CA46, JEKO-1, HMy2.CIR and NALM6 all expressed cell surface CD22 and the percentage of positivity was 100%. CD22 was also expressed on the surface of primary tumor cells in 2 cases, and the positivity rate was also 100%. As a control, the acute myeloid leukemia cell line MOLM13 cells did not express CD22 on the surface (Fig. 1B,C).

To investigate the effect of Chidamide on cells, the cell lines and the primary tumor cells were treated with different concentrations of Chidamide (0.25–0.5–1–2–4–8 μM) for 72 h and dose-dependent proliferation inhibition was observed with negligible inhibition of proliferation at the concentration of 0.5 μM (Supplementary Fig. S1A). Then each cell line and primary cells were treated with 0.25 μM and 0.5 μM of Chidamide in order not to affect the proliferation of cells, and apoptosis was detected at different time points (3–6–9–12 days). The results showed there was no significant apoptosis induced by 0.25 μM and 0.5 μM Chidamide up to 12 days (Supplementary Fig. S1B). After that, we tested the impact of Chidamide on CD22 expression in eight cell lines and two cases of primary cells. All cells demonstrated an increase in CD22 MFI upon exposure to Chidamide for 72 h at 0.25 μM or 0.5 μM in vitro and a dose-dependent manner was observed with the effect of Chidamide at 0.5 μM more obvious (Supplementary Fig. S1C). The cells exposure to 0.5 μM Chidamide were centrifuged and fresh medium was replaced containing 0.5 μM Chidamide every 3 days, and the expression of CD22 was detected intermittently for four times (3–6–9–12 days). The elevation of CD22 MFI was most remarkable on day 6 and diminished on day 12 (Fig. 1D).

### Chidamide enhances the functionality of CD22 CAR T Cells in vitro by upregulating cell surface CD22 expression

**Figure 2:**
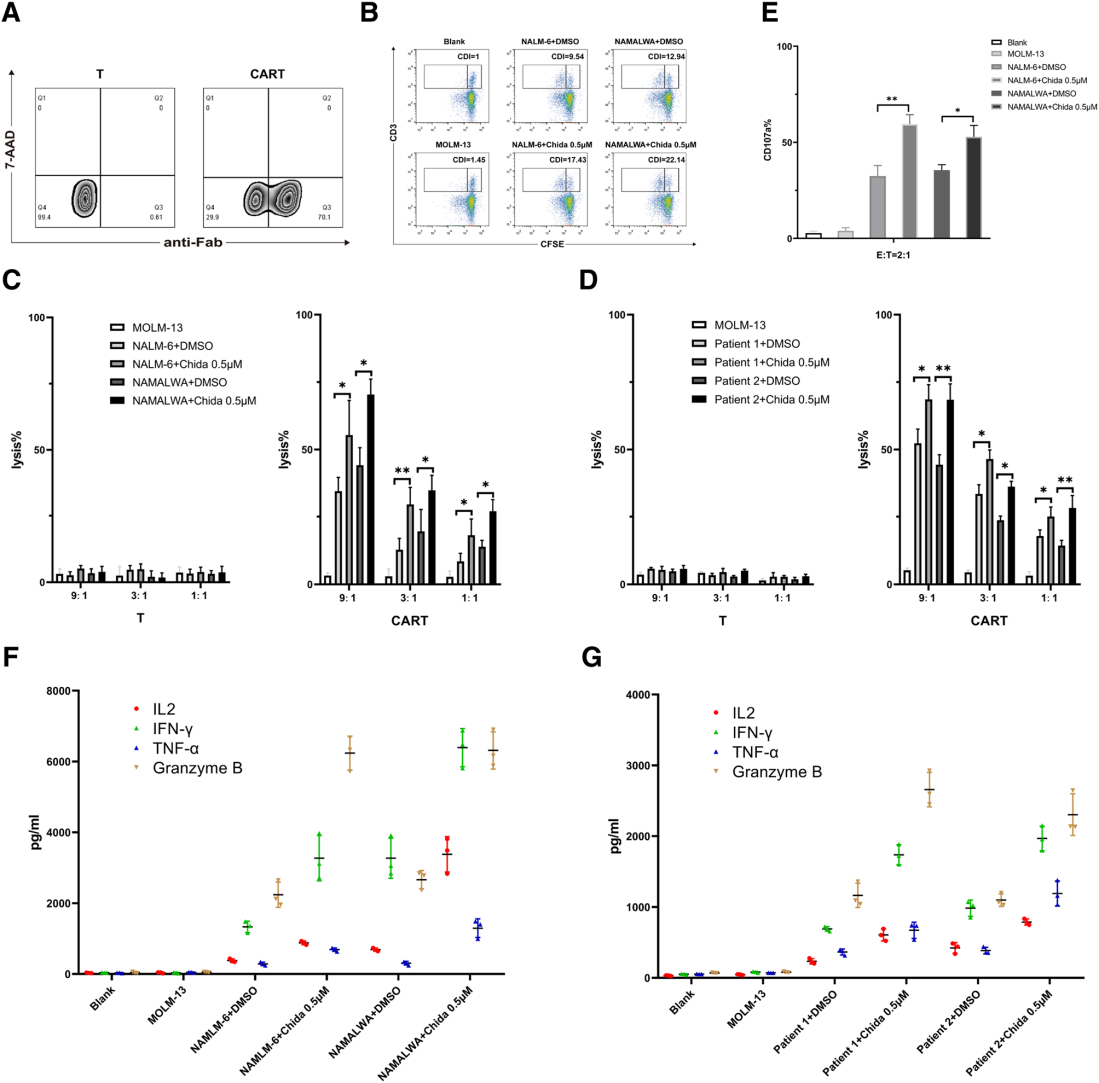
Chidamide enhances the functionality of CD22 CAR T Cells in vitro by upregulating cell surface CD22 expression. (**A**) Transfection rate of T cells expressing CAR22. (**B**) The expansion of CAR T cells was evaluated by CFSE. CAR T cells were stained with CFSE and co-cultured with different target cells. When conducting flow cytometry, 5000 parental cells in the right square were collected and the left square represented the progeny cells. CDI was calculated using the group of blank as a control. (**C**–**D**) Different target cells (MOLM13, NALM6 pretreated with DMSO or Chidamide, NAMALWA pretreated with DMSO or Chidamide, and primary cells pretreated with DMSO or Chidamide) in cytotoxicity assays at various effector: target cell ratios. Here, we show the combined results of three independent experiments. After CAR T cells were co-cultured with different target cells, the degranulation levels (**E**) were detected by flow cytometry and cytokines were measured by ELISA from cell culture supernatants (**F**–**G**). Statistics were calculated using unpaired t-test.

We successfully generated CAR-T cells targeting CD22 and CAR expression was detected 6 days after lentivirus transfection (Fig. 2A). To assess the functionality of CAR T cells in vitro, NAMALWA and NALM6 pretreated with 0.5 μM Chidamide or control DMSO for 3 days were selected as CD22-positive target cells and MOLM13 as CD22-negative target cells. The results showed that expansion of CAR T cells co-cultured with CD22-positive target cells was much more notable compared with those co-cultured with CD22-negative target cells, and target cells exposured to Chidamide before stimulated CAR T cell proliferation more significantly (Fig. 2B). Then the cytotoxicity of CAR T cells on different target cells was detected. In different ratios of effector versus target, CD22 CAR T cells could specifically lyse CD22-positive target cells, and when comparing cells pretreated with DMSO, the cells exposure to Chidamide elicited more stronger cytotoxicity of CAR T cells (*P* < 0.05) (Fig. 2C). The same cytotoxicity assays were performed in primary tumor cells and the result was consistent with in cell lines (Fig. 2D). In addition, CD22 CAR T cells specifically degranulated and secreted cytokines including IL-2, TNF-α IFN-γ and Granzyme B when co-cultured with CD22-positive target cells, and CAR T cells degranulated more notably (*P* < 0.05) when co-cultured with Chidamide-pretreated target cells compared with DMSO-pretreated target cells (Fig. 2E), with four cytokines secreted much more higher (*P* < 0.05) (Fig. 2F). The similar cytokine assay experiments were also conducted in primary cells and the results showed consistency with the cell lines (Fig. 2G).

### Exposure to Chidamide has no significant affect on the function of CAR T cells

To verify the effect of Chidamide itself on CAR T cells, dose-dependent proliferation inhibition by Chidamide for 72 h was also observed on CAR T cells (Supplementary Fig. S2A) and the concentrations of 0.25 μM and 0.5 μM were selected to conduct the subsequent detection. It was observed that Chidamide itself did not influence the phenotype of CAR T cells (Supplementary Fig. S2B) as well as their degranulation (Supplementary Fig. S2C) or cytotoxicity (Supplementary Fig. S2D).

### Chidamide increases CD22 antigen expression and bolsters antitumor effects of CAR T cells in vivo

**Figure 3:**
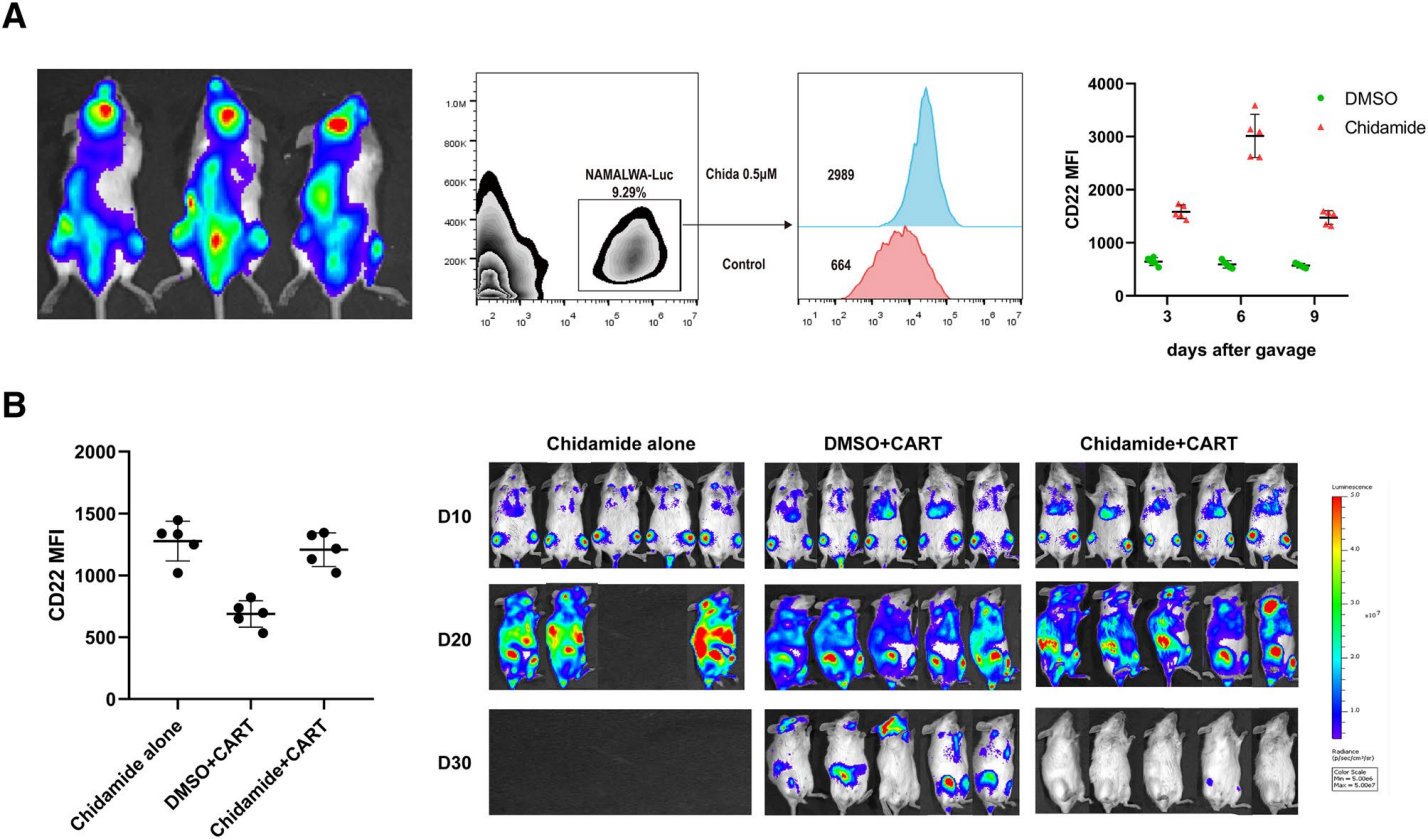
Chidamide increases CD22 antigen expression and bolsters antitumor effects of CAR T cells in vivo. (**A**) A NAMALWA-xenograft mice model was constructed and the GFP + Luciferase + NAMALWA cells were detected in peripheral blood. When given by orally administrated with Chidamide at a final concentration of 3 mg/kg for 3 days, the MFI of CD22 on the surface of tumor cells was measured at days 3, 6 and 9 after gavage. (**B**) NSG mice were injected with GFP + Luciferase + NAMALWA cells on Day 0, DMSO control and Chidamide was orally administrated at day 10 for 3 days and CD22 CAR T cells were injected on Day 13 after detecting the MFI of CD22 on the surface of tumor cells. Leukemia progression was monitored using IVIS technology and luciferin-D IP injections.

To evaluate the impact of Chidamide-mediated increase in CD22 site density on in vivo CAR functionality, a NAMALWA-xenograft mice model was constructed and the peripheral blood of mice was detected in day 10 to confirm there being tumor cells in blood. Then Chidamide was given by gavage at a final concentration of 3 mg/kg on day 10, and then the apoptosis and the site density of CD22 on the surface of tumor cells in peripheral blood were measured by orbital blood collection intermittently after successive 3 days of gavage. The results showed that the in vivo tumor cells did modulate CD22 expression in response to Chidamide and the effect of upregulation for CD22 expression was most significant at day 6 after the first gavage but could last for 9 days (Fig. 3A). In addition, there was no significant differences in apoptosis induced by Chidamide compared with DMSO up to 9 days (Supplementary Fig. S1B).

Base on above, we tested the administration of Chidamide as a "priming therapy" prior to CAR T cells infusion. Mice were gavage with Chidamide at a final concentration of 3 mg/kg on day 10 for consecutive 3 days. On day 13, the site density of CD22 on the surface of tumor cells in peripheral blood was measured and CAR T cells were injection at a dose of 1 × 10^7^ cells. The group of mice treated with Chidamide presented higher CD22 expression in tumor cells, consistent with the previous. The first group of mice orally administrated with Chidamide alone died at a short time; the tumor could not be completely cleared in the second group injected with CAR T cells alone; and in the third group, CAR T emerged remarkable antitumor efficacy with the tumor eliminated completely (Fig. 3B).

### Chidamide-mediated surface CD22 upregulation may occur via distribution of CD22 rather than increased CD22 gene expression

**Figure 4:**
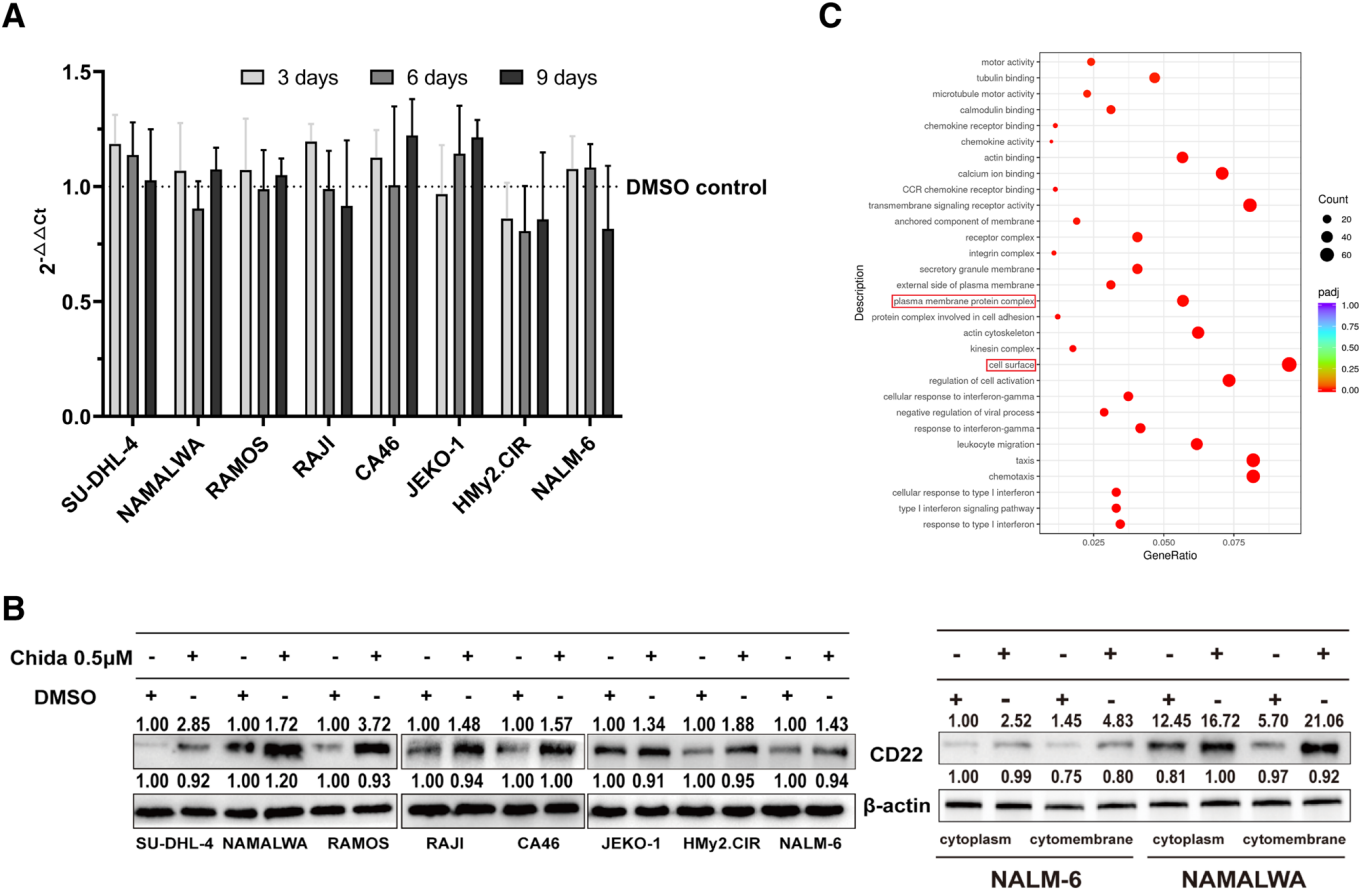
Chidamide-mediated surface CD22 upregulation may occur via distribution of CD22 rather than increased CD22 gene expression. (**A**) CD22 mRNA expression of Eight cell lines co-incubated with 0.5 μM Chidamide were harvested on days 3, 6 and 9 and their CD22 mRNA expression was analyzed using RT-PCR. (**B**) NALM6 and NAMALWA were co-incubated with 0.5 μM Chidamide for 72 h and the total CD22 protein as well as cytosolic and cytoplasmic CD22 proteins were detected by Western Blotting. (**C**) NALM6 and NAMALWA co-incubated with 0.5 μM Chidamide for 72 h were harvested and a transcriptome sequencing analysis was conducted.

To investigate the mechanism of upregulation of cell surface CD22 expression by Chidamide, eight cell lines treated with 0.5 μM Chidamide were harvested on days 3, 6 and 9, and their CD22 mRNA expression were discovered to have no significant differences (Fig. 4A). But in contrast, when exposed to 0.5 μM Chidamide for 72 h, the total CD22 protein in all cell lines had elevated significantly. In addition, the cytosolic and cytoplasmic proteins from NALM6 and NAMALWA cells were extracted and it was found that the upregulation of CD22 protein expression in membrane was more significant than in cytoplasm (Fig. 4B).

Finally, we conducted a transcriptome sequencing analysis of NAMALWA and NALM6 cells pretreated with 0.5 μM Chidamide or control DMSO for 72 h. Using Gene Set Enrichment Analysis (GSEA) of DEGs in gene ontology (GO), Chidamide-modulated genes in both cell lines were categorized into multiple pathways with genes related to cell surface the most notable (Fig. 4C).

## Discussion

CAR T immunotherapy has provided more therapeutic options for patients with refractory/relapse B-cell malignancies, but an increasing number of research has shown that the target density will affect the efficacy of CAR T^22,23^, and the reduction or loss of target antigen is also an important rationale of short remission duration after CAR T therapy^8,9^. As advancement of epigenetics, more and more epigenetic modulating drugs are being applied in the clinic, and their effects on the expression of different proteins have been reported in many previous studies^17–19^. In our study, we combined the epigenetic modulating drug HDACi Chidamide with CAR T immunotherapy for the first time and verified that Chidamide is able to enhance the efficacy of CD22 CAR T by upregulating CD22 expression on the surface of tumor cells in vitro and in vivo, providing a preclinical evidence to support the use of drug-mediated antigen density modulation to overcome such limitations for CAR T therapy. Our data has confirmed two important findings: (A) Chidamide promotes the function of CAR T cells by specifically upregulating target expression on the surface of B-cell tumor cells in vitro and in vivo without affecting the function of T cells by itself, and (B) Chidamide may affect CD22 protein transportation and distribution through modulation of post-transcriptional modifications thus upregulate the expression of CD22 on the cell surface.

We first validated the effect of Chidamide on surface CD22 expression in vitro and in vivo. To eliminate the effects of cell proliferation and apoptosis, we determined the appropriate concentration of Chidamide for subsequent experiments. Similar to the upregulation of cell surface CD20 expression by Entinostat, another HDACi^17^, Chidamide significantly elevated surface CD22 expression in eight involved cell lines, with a more than twofold increase in MFI; however, in contrast to previous research, Chidamide did not impact cell surface CD20 expression^18^ (data not shown) and Decitabine, another epigenetic regulatory drug, did not affect cell surface CD22 expression (data not shown), suggesting that the modulation of cell surface CD22 expression by Chidamide is specific and may be related to the regulation of CD22 being modulated by multiple factors^24^. It has been confirmed that CD22 molecules on the surface of B lymphocytes are internalized into cells via lattice-protein-dependent endocytosis, and in addition to being degraded in lysosomes, a part of them are recycled back to the cell membrane via intracellular circulating endosomal structures and secreted out of cells with exosomes^25–28^. Assuming the corresponding transporter proteins are regulated by post-transcriptional modifications, it will lead to changes in the intracellular transportation and redistribution for CD22, which may alter the site density on the cell surface.

We next assessed the effect of Chidamide on CAR T functionality. To eliminate the effect of Chidamide itself on CAR T cells, we first treated CAR T cells with Chidamide and detected their functions, and found that Chidamide did not affect CAR T cell phenotype, degranulation, or antitumor efficacy in vitro. Subsequent experiments confirmed that Chidamide enhanced the function of CD22 CAR T both in vitro and in vivo by upregulating target antigen density, which was consistent with the findings that antigen density affects CAR T cell function as reported in the previous studies^12,13^. Our study did not focus on the threshold of antigen density required to activate CAR T cells, but substantiated that increased antigen density promotes CAR T functionality. For the two target cells NAMALWA and NALM6 with different initial antigen densities as well as primary cells, the degree of elevation in antigen density promoted by Chidamide was not identical. However, the effect of Chidamide in enhancing CAR T efficacy on these target cells was approximately the same suggesting that the antigen density required to activate CAR T cells may not be the same in different cells, but the conclusion that increased antigen density promotes CAR T functionality is still reliable.

Related research has shown that in patients who relapsed due to diminished tumor CD22 expression after CD22 CAR T therapy, whole-exome sequencing and transcriptome sequencing of tumor cells revealed no mutations in the exon region of the CD22 gene and no new CD22 variant spliceosomes, with CD22 mRNA expression upregulated, suggesting that CD22 expression alterations occurred in a post-transcriptional regulatory process. This is also consistent with the findings of our study. In addition, we found that the most significant differences between groups were enriched in cell surface-related genes and plasma membrane protein complex-related genes by transcriptome sequencing, further indicating that Chidamide may affect cell surface protein distribution and thus upregulate cell surface CD22 expression through post-transcriptional modifications.

However, our study leaves something to be desired. We verified that upregulation of CD22 expression could enhance the function of CD22 CAR T, but did not determine the target antigen density that can activate as well as enhance the efficacy of CAR T. The antigen density may vary in different cells, which needs to be explored in further research. Moreover, in terms of mechanism, our study only superficially revealed that the modulation of cell surface CD22 by Chidamide occurs by post-transcriptional translational modifications, but the specific pathways involved has not been investigated in more detail, which could not forcefully explain this appearance we discovered.

In conclusion, we established that Chidamide can specifically upregulate CD22 expression on the surface of B-cell tumor cells in vitro and in vivo, thus promoting the efficacy of CD22 CAR T; and the mechanism may be related to that Chidamide promotes histone acetylation by inhibiting histone deacetylase, further regulating post-transcriptional modifications, affecting the transportation and redistribution of CD22 protein and thus increasing cell surface CD22 expression. Our study provides a reliable rationale for the clinical combination of Chidamide and CD22 CAR T immunotherapy, which may enhance the efficacy of CAR T through eliminating the limitations of CAR T resulted by reduction or loss of target antigen and contribute to CAR T immunotherapy!

## Materials and methods

### Cell lines and primary cells

Diffuse large B-cell lymphoma cell line SU-DHL-4, Burkitt lymphoma cell lines NAMALWA, RAMOS, RAJI and CA46, mantle cell lymphoma cell line JEKO-1, B lymphoblastic lymphoma cell line HMy2.CIR, B-ALL cell line NALM6, acute myeloid leukemia cell line MOLM13 were purchased from ATCC or DSMZ and preserved in our lab and all verified before being used in following experiments. All cell lines were cultured with RPMI 1640 medium containing 10% fetal bovine serum (FBS, Gibco, USA) in humidified 5% CO_2_ at 37 °C. Primary human tumor cells were obtained by magnetic bead sorting from peripheral blood of 2 clinical patients who suffered relapse after CD19 CART treatment, 1 case of B-NHL and 1 case of B- ALL. We confirm that all methods were carried out in accordance with relevant guidelines and regulations or in accordance with the Declaration of Helsinki. We confirm that informed consent was obtained from all subjects and/or their legal guardian(s). And we confirm that all experimental protocols were approved by a named institutional and/or licensing committee.

### Generation of human CD22 CAR T cells

Lenti-X™293 T cells were co-transfected with the vector encoding CD22 CAR, and the viral supernatants were collected 72 h later. The lentiviral concentrate was collected by ultracentrifugation and then aliquoted and stored at − 80 °C. Peripheral blood samples were taken from healthy blood donors, and the T cells were separated from PBMCs using CD3 microbeads (Miltenyi Biotec GmbH, Bergisch Gladbach, Germany) following the manufacturer's instructions. Then, cells were stimulated with Dynabeads™ Human T-Activator CD3/CD28 (Gibco, Grand Island, NY, USA) at a 1:1 ratio in CTS™ OpTmizer™ medium (Gibco, Grand Island, NY, USA) containing 2 mM l-glutamine (Gibco, Grand Island, NY, USA) and 200 IU/mL rhIL-2 (PeproTech, Rocky Hill, NJ, USA). Within 24 h, the T cells were transfected with concentrated lentivirus at a multiplicity of infection (MOI) ranging from 3 to 5. Then, the T cells were cultured at a density of 5 × 10^5^ to 1 × 10^6^/mL.

### NAMALWA xenograft in vivo studies

The functionality of CD22 CAR T cells were evaluated in vivo in NAMALWA xenograft with NCG mice (Gempharmatech, Nanjing, China) aged 6–8 weeks. The female mice received 1 × 10^6^ GFP + Luciferase + NAMALWA cells intravenously on day 0. 7 days later, the mice were divided into three groups and were intravenously injected with CD22-CAR-transduced T cells at the indicated quantity. To monitor leukemia burden, luciferin-D (YEASEN, Shanghai, China) was injected into mice intraperitoneally and imaged 8 min later using In Vivo Imaging System (IVIS) technology (Caliper Life Sciences, USA). Bioluminescent signal flux (luminescence) for mice was measured using Living Image Version 4.1 software. All animal experiments were approved by the Institutional Committee of Animal Care of Tongji Medical College, Huazhong University of Science and Technology, Wuhan, China. All experiments were performed at the animal experimental center of Tongji Medical College. All methods were carried out in accordance with relevant guidelines and regulations and reported in accordance with ARRIVE guidelines.

### Chidamide treatment

Chidamide was purchased from Med Chem Express (USA), dissolved in Dimethyl sulfoxide (DMSO) at the recommended concentration according to the instructions, and stored at − 80 °C. For vitro experiments, Chidamide was administered to cells at the indicated concentration, and the cells were centrifuged and replaced with fresh medium containing the corresponding concentrations of Chidamide every 3 days, while the control group was treated with DMSO at an equal volume. For tumor/T cells pretreatment, Chidamide was administered to cells at a indicated concentration, DMSO vehicle control was administered to control-treated cells at an equal volume. Prior to co-culture, Chidamide- and DMSO- treated cells were washed three times in sterile PBS. For all in vivo experiments, Chidamide was diluted in DMSO, PEG300, Tween80 and saline in turn and orally administered at 3 mg/kg (30 µg/20 g mouse). For control-treated groups, an equal volume of DMSO was diluted and administered in the same manner.

### Flow cytometry

FACS analysis of cell surface CAR and protein expression was performed using a CytoFLEX S flow cytometer (Beckmam Coulter, CA, USA). CD22-CAR was detected by incubation with APC-F(ab)2 (Jackson Immunoresearch, USA). The following human monoclonal antibodies were used for detection of cell surface proteins: CD22-APC, CD22-PE, CD19-PE, CD45-PerCP/Cy5.5, CD3-APC/Cy7, CD8-Pacific Blue, CD4-APC, CD45RO-PE/Cy7, CD45RA-FITC, CCR7-APC (all from BioLegend). CD22 site density was quantified by Quantity-PE beads (BD Biosciences, USA) following instructions. Both GFP-expressing cells and CFSE dye-labeled cells were identified through the FITC channel.

### Proliferation and apoptosis assays

Cell lines and primary cells were assayed for cell proliferation using the CCK8 kit (Vazyme Biotech, Nanjing, China), and cell proliferation inhibition rate (%) = (OD treated − OD blank)/(OD control − OD blank) × 100%. Apoptosis was detected by AnnexinV/7-AAD apoptosis assay kit (BD Biosciences, USA), and the apoptosis rate was detected by flow cytometry after cell staining. The proliferation of T cells was detected by CFSE. CD22-CAR-transduced T cells and mock-transduced T cells were labeled with CFSE separately and co-cultured with different target cells at a 1:1 ratio in 24-well plates with IL-2-free T cell complete medium (TCM). 4 days later, Cells were stained with CD3-APC/Cy7, and the cell division index (CDI) was calculated to compare the proliferation differences of T cells in each group.

### CD107a release assay

The cell fractions positive for the CD22 CAR were adjusted to 30% by adding untransfected T cells. CAR T cells were washed to remove IL-2 and resuspended in RPMI medium and then were co-cultured with different target cells at a 2:1 ratio at 37 ºC in an incubator. CD107a-PE/Cy7 antibody (BioLegend, CA, USA) was initially added at 20 µL/mL. One hour after incubation, monensin (Golgi-Stop, BD Biosciences, NJ, USA) was added to a final concentration of 6 mg/mL, and incubation was allowed to last an additional 3 h. The cells were then stained with CD3-APC (BioLegend, CA, USA) and CD8-Pacific Blue (BioLegend, CA, USA) antibodies, and CD107a expression on the CD3 and CD8 double-positive cells was detected.

### In vitro cytotoxicity

The calcein release assay was performed to detect the cytotoxicity of CAR T cells in vitro. The target cells (NALM6 and NAMALWA cells pretreated with Chidamide, NALM6 and NAMALWA cells pretreated with DMSO) positive for CD22 and target cells (MOLM13 cells) negative for CD22 were labeled with calcein (Aladdin, Shanghai, China) and then co-cultured with effector cells (untransfected T cells and CAR22-transfected T cells) in 96-well plates at different ratios (9:1, 3:1, and 1:1). Wells with co-cultured target cells and PBS served as spontaneous release wells, and wells with co-cultured target cells and lysis solution were taken as maximum release wells. The cultures were centrifuged 3 h after incubation, and the supernatants were transferred to another 96-well plate. The fluorescence of each well (*F*) was measured on a microplate reader, and the tumor-killing efficiency was calculated according to the following formula: lysis (%) = (*F* experimental wells − *F* spontaneous release)/(*F* maximal release − F spontaneous release) × 100%.

### Detection of intracellular cytokines

As in the CD107a assay, different effector cells (untransfected T cells and CAR22-transfected cells) were co-cultured with different target cells (NALM6 and NAMALWA cells pretreated with Chidamide, NALM6 and NAMALWA cells pretreated with DMSO, and MOLM13 cells) at a 2:1 ratio in 96-well plates and incubated at 37 °C for 24 h. Plates were spun at 1200 RPM for 6 min to pellet the cells, and cytokine concentrations in the culture supernatants were measured using ELISA kit (NEOBIOSCIENCE, China), including IL-2, TNF-α, IFN-γ and GranzymeB following the manufacturer's instructions.

### Quantitative real-time polymerase chain reaction analysis

The appropriately treated cells (SU-DHL-4, NAMALWA, RAMOS, RAJI, CA46, JEKO-1, HMy2.CIR, and NALM6 cells) were collected and resuspended in Trizol agent for RNA extraction. cDNA was converted from 1 μg RNA using a reverse transcriptase kit (Vazyme Biotech, Nanjing, China). Gene expression was assessed using qRT-PCR following the instructions and the obtained data were analyzed by the 2^−ΔΔCt^ method.

### Western blotting analysis

Cytoplasmic proteins, cytosolic proteins and total proteins were extracted using a protein extraction kit (abcam, UK) and proteins in all samples were quantified with Bicinchoninic acid protein assay. Proteins of equal amounts from all samples were separated with SDS/PAGE gel (Bio-Rad, USA) and transferred onto PVDF membrane (Bio-Rad, USA). Bands were sealed with 5% skim milk, incubated in primary antibodies (anti-CD22 and anti-GAPDH, CTS, SUA) at 4 °C overnight, then in secondary antibodies (HRP-goat anti-rabbit IgG, CTS, SUA) at room temperature for 1 h, and then examined and analyzed by using ChemiDoc™ XRS + with Image Lab™ Software (Bio-Rad, USA). Due to the significant difference of molecular weight between the reference protein and the target protein as well as the inconsistent conditions for transferring onto PVDF membrane, the gels were cut before transferring, and the incubation of the antibody as well as exposure of the reference protein and the target protein were also carried out separately.

### Transcriptome sequencing and data analysis

Three biological replicates RNA samples were collected From NAMALWA and NALM6 cell lines which were grouped as control or treated with 0.5 μM Chidamide for 3 days. Total RNA was used for RNA-seq analysis. cDNA library construction and sequencing were performed by Beijing Genomics Institute using BGISEQ-500 platform. High-quality reads were aligned to the human reference genome (GRCh38) using Bowtie2. The levels of expression for each of the gene were normalized to fragments per kilobase of exon model per million mapped reads (FPKM) using RNA-seq by Expectation Maximization (RSEM)46. Pathway analysis was performed using the GSEA software developed by the Broad Institute47. The significance of DEGs was confirmed with the BGI bioinformatics service using the combination of the absolute value of log2-ratio ≥ 1 and P ≤ 0.05 in this research.

### Data analysis

SPSS 25.0 software (IBM, NY, USA) was used for data analysis. Comparison of quantitative data between groups was performed using Student's t test, one-way ANOVA, or a nonparametric test. *P* < 0.05 was considered statistically significant (*P < 0.05; **P < 0.01; ***P < 0.001), and various statistical graphs were used. Were plotted using Graphpad Prism 8.0 software.

## Supplementary Information

Supplementary Legends.

Supplementary Figure S1.

Supplementary Figure S2.

Supplementary Figure S3.

## Data Availability

The data that support the findings of this study are available from the corresponding author upon reasonable request.
