# Supplementary material for: Upregulation of CD22 by Chidamide promotes CAR T cells functionality
Source: Sci Rep. 2021 Oct 19;11:20637. doi: 10.1038/s41598-021-00227-4 (PMC8526578; doi:10.1038/s41598-021-00227-4)
Supplement: Supplementary file 1 — Supplementary Legends. [file 41598_2021_227_MOESM1_ESM.docx]

**Supplementary Information**

**Upregulation of CD22 by Chidamide promotes CAR T cells functionality**

Xin Yang^1^, Qiuxia Yu^1^, Hao Xu^1^, Jianfeng Zhou^1^

^1^ Department of Hematology, Tongji Hospital, Tongji Medical College, Huazhong University of Science and Technology, Wuhan, Hubei, China.

*Correspondence to:* Hao Xu, Department of Hematology, Tongji Hospital, Tongji Medical College, Huazhong University of Science and Technology, NO.1095 Jie Fang Avenue, Wuhan 430030, Hubei, China; Tel: +8618986096730, E-mail: zyxuhao@163.com

**Titles and captions to supplementary figures**

**Supplementary Fig.1** The effect of Chidamide on cell lines and primary cells. (A) Eight cell lines and two primary tumor cells were co-incubated with different concentrations of Chidamide and proliferation inhibition was analyzed using kit at 72 h after Chidamide exposure. (B) Eight cell lines and two primary tumor cells were co-incubated with 0.25 μM or 0.5 μM Chidamide and apoptosis was detected by flow cytometry at different time points (3-6-9-12 d); the NAMALWA-xenograft mice were given DSMO or Chidamide by gavage on day 10, and the detection of apoptosis of tumor cells in peripheral blood was performed at different time points (3-6-9 d) after successive 3 days of gavage along with the detection of CD22 expression. (C) Eight cell lines and two primal tumor cells were co-incubated with 0.25 μM or 0.5 μM Chidamide and CD22 MFI was analyzed using flow cytometry at 72 h after Chidamide exposure.

**Supplementary Fig.2** Exposure to Chidamide has no significant affect on the function of CAR T cells. (A) CAR T cells were co-incubated with different concentrations of Chidamide and proliferation inhibition was analyzed at 72 h after Chidamide exposure. (B) CAR T cells were co-incubated with 0.25 μM or 0.5 μM Chidamide and the phenotype of CAR T cells was detected by flow cytometry at 72 h after Chidamide exposure. (C) CAR T cells pretreated with DMSO, 0.25 μM or 0.5 μM Chidamide were co-incubated with different target cells (MOLM13 and NALM6), and the degranulation levels were measured by flow cytometry. (D) CAR T cells with different pretreatment were co-incubated with different target cells in cytotoxicity assays at various effector: target cell ratios. Statistics were calculated using unpaired t-test.

**Supplementary Fig.3** The original, unprocessed versions of images for Western Blotting.
