# Supplementary figures and images for: Upregulation of CD22 by Chidamide promotes CAR T cells functionality
Source: Sci Rep. 2021 Oct 19;11:20637. doi: 10.1038/s41598-021-00227-4 (PMC8526578; doi:10.1038/s41598-021-00227-4)

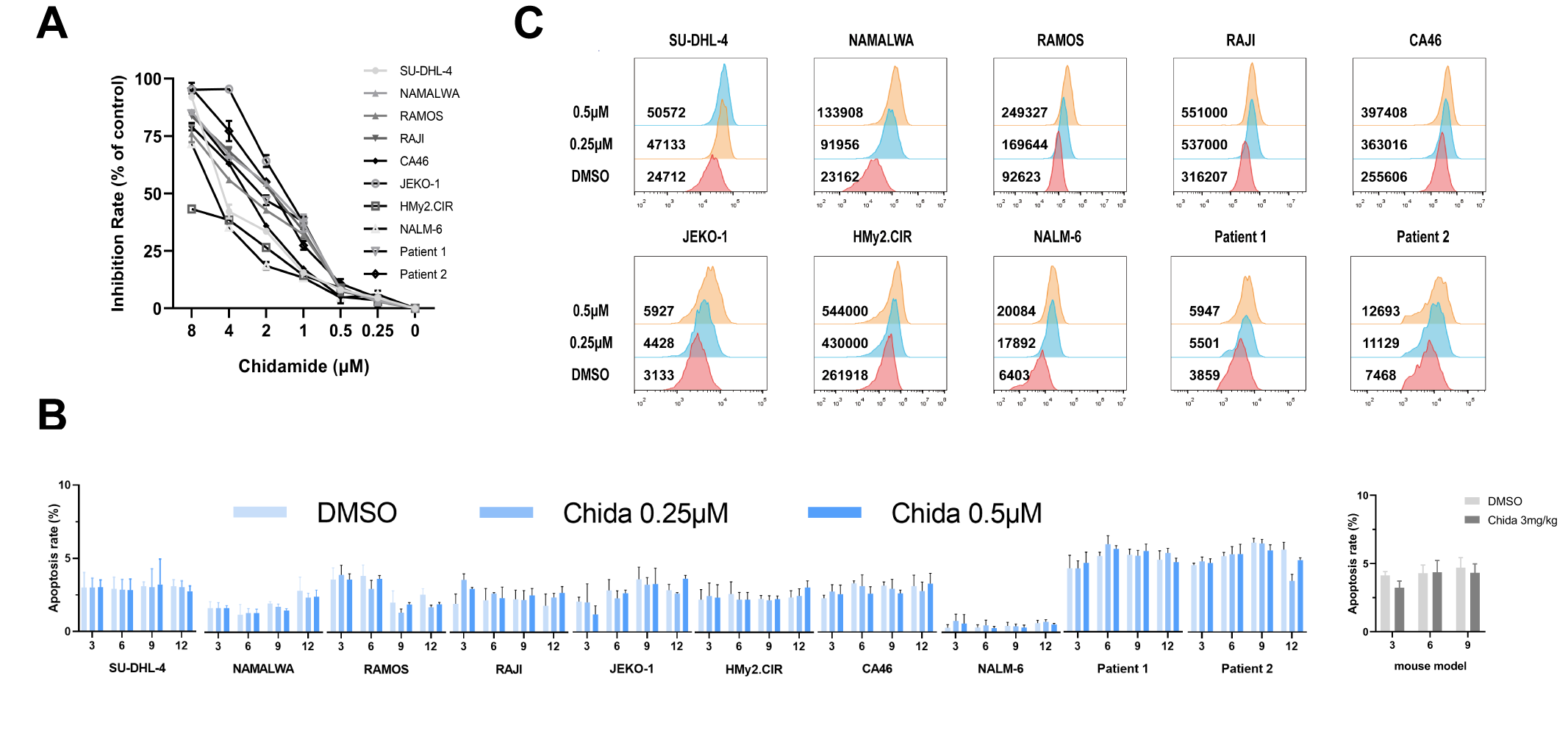

Supplement: Supplementary file 2 — Supplementary Figure S1. [file 41598_2021_227_MOESM2_ESM.tif]

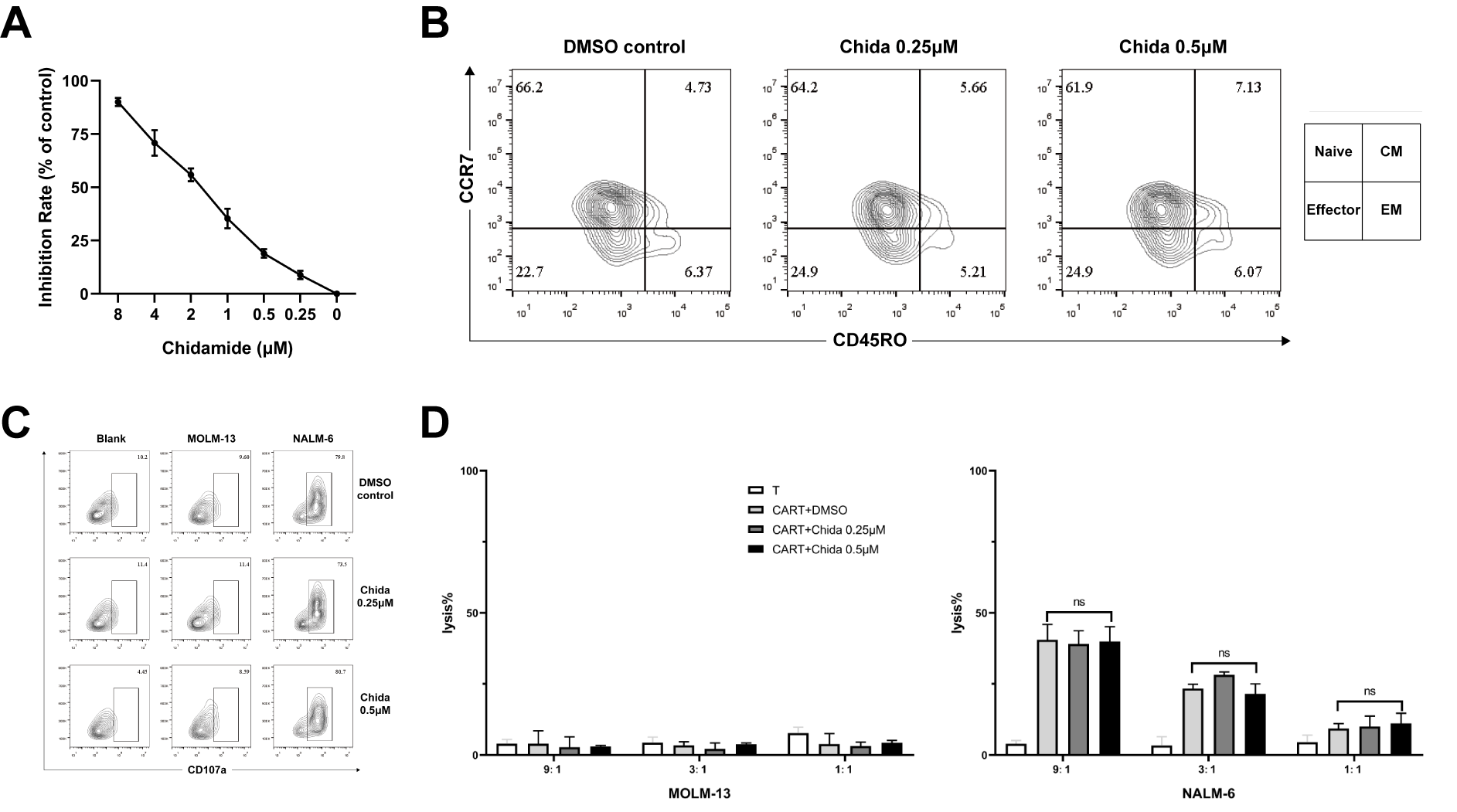

Supplement: Supplementary file 3 — Supplementary Figure S2. [file 41598_2021_227_MOESM3_ESM.tif]

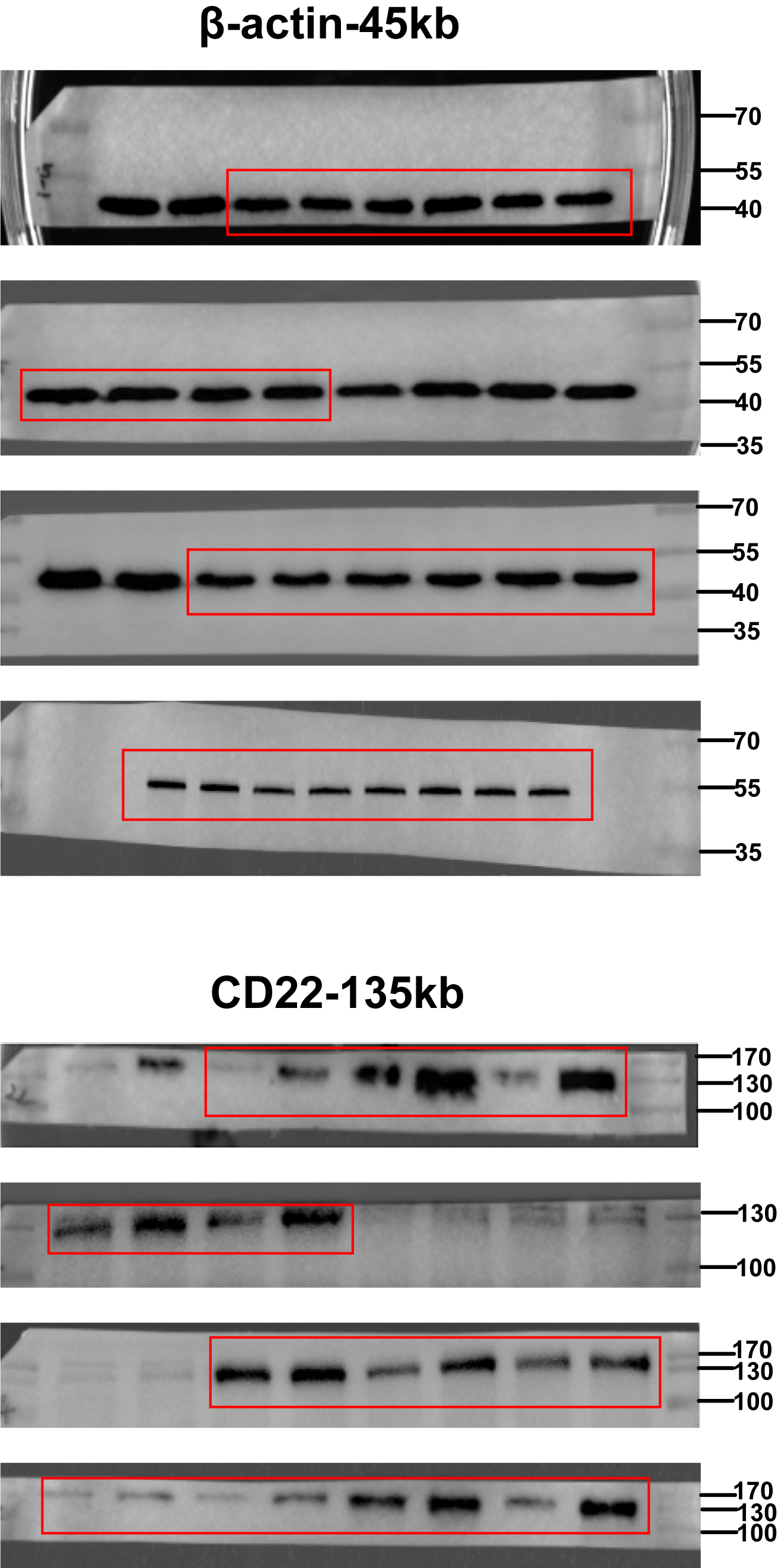

Supplement: Supplementary file 4 — Supplementary Figure S3. [file 41598_2021_227_MOESM4_ESM.tif]
